# Quantifying the impacts of defaunation on natural forest regeneration in a global meta-analysis

**DOI:** 10.1038/s41467-019-12539-1

**Published:** 2019-10-14

**Authors:** Charlie J. Gardner, Jake E. Bicknell, William Baldwin-Cantello, Matthew J. Struebig, Zoe G. Davies

**Affiliations:** 1 Durrell Institute of Conservation and Ecology (DICE), School of Anthropology and Conservation, University of Kent, Canterbury, Kent CT2 7NR UK; 2 WWF UK, The Living Planet Centre, Brewery Road, Woking, GU21 4LL UK

**Keywords:** Ecosystem services, Forest ecology, Biodiversity

## Abstract

Intact forests provide diverse and irreplaceable ecosystem services that are critical to human well-being, such as carbon storage to mitigate climate change. However, the ecosystem functions that underpin these services are highly dependent on the woody vegetation-animal interactions occurring within forests. While vertebrate defaunation is of growing policy concern, the effects of vertebrate loss on natural forest regeneration have yet to be quantified globally. Here we conduct a meta-analysis to assess the direction and magnitude of defaunation impacts on forests. We demonstrate that real-world defaunation caused by hunting and habitat fragmentation leads to reduced forest regeneration, although manipulation experiments provide contrasting findings. The extirpation of primates and birds cause the greatest declines in forest regeneration, emphasising their key role in maintaining carbon stores, and the need for national and international climate change and conservation strategies to protect forests from defaunation fronts as well as deforestation fronts.

## Abstract (web summary)

The defaunation of vertebrates may disrupt forest functioning through the loss of plant-animal interactions, but impacts on forests remain unquantified. Here the authors show that seed dispersal is a key interaction and defaunation of primates and birds negatively impacts forest regeneration.

## Introduction

Intact forests provide irreplaceable ecosystem services, including regulation of weather regimes and carbon storage, but their functioning depends on the maintenance of ecological communities^1^. However, human activities such as habitat conversion, degradation and hunting are causing vertebrate range contractions, population declines and extinctions at local and global scales, and thus altering faunal communities and their interactions with forest flora. For example, 41% of monitored tropical forest vertebrate species populations (*n* = 369) declined between 1970 and 2012^2^, and unsustainable hunting is thought to occur in a greater proportion of remaining forests than all other degradation drivers^1^. This vertebrate defaunation^3,4^ particularly affects larger-bodied species because they are readily targeted for food and are both ecologically and demographically vulnerable^5,6^. As a result, there are now many ‘empty forests’ around the world, which are essentially devoid of large- and medium-sized mammals^7,8^.

Defaunation is expected to have serious consequences for forest functioning and tree community composition through the disruption of the woody vegetation-animal interactions that influence forest regeneration, such as seed predation^9^, herbivory^10–12^, pollination^13,14^ and seed dispersal. Seed dispersal is likely to be particularly impacted because more than 80% of tropical forest woody plants produce vertebrate-dispersed seeds^15,16^ and, in general, the dispersal services provided by large vertebrates are non-redundant, meaning that they cannot be substituted by other species^17–19^. Large-bodied vertebrates are the only group to perform the important role of long-distance dispersal of large seeds^10,20^.

An improved comprehension of the consequences of defaunation on natural forest functioning is crucial for informing and targeting climate change and conservation policy developments at national and international scales. In particular, an enhanced understanding is needed because present forest regeneration will ultimately determine the composition of future forests and, therefore, the ecosystem services they provide. Nonetheless, the current evidence-base consists of modelled scenarios and a body of empirical studies that are yet to be synthesised collectively.

**Fig. 1:**
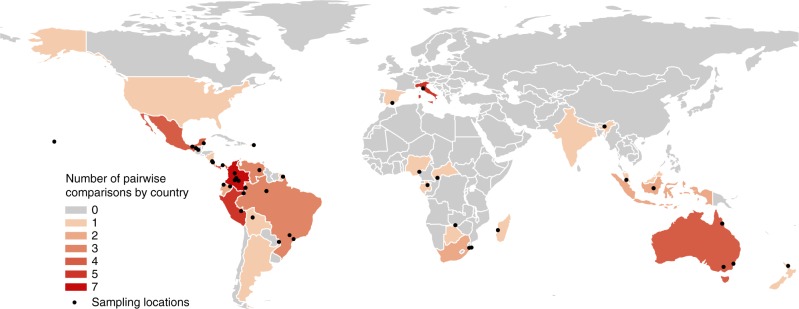
Map of study sites by country and study location. Graded colours illustrate the number of pairwise comparisons of woody vegetation outcome responses between high fauna and low fauna treatments per country, and points indicate the sampling locations

Here, we conduct a meta-analysis to measure the varied impacts of vertebrate defaunation on the woody components of forests globally. We focus on vertebrates because, in addition to their interactions with plants, they are the most extensively studied faunal group, they account for a large proportion of forest faunal biomass^5^, and they are suffering declines throughout the world’s forests^2^. Through a systematic literature search we identified 43 papers (Supplementary Table 1) that recorded measures of forest woody vegetation or regeneration in multiple treatments that differ in terms of the abundance, density or richness of vertebrates. The papers measured defaunation impacts on a range of woody vegetation outcome response variables, including the density or richness of the regenerating tree community, the density or dispersion of individual tree species, woody vegetation cover, and biomass. Overall, 92% of the extracted data measured impacts on regenerating cohorts of tree seedlings and saplings, with the remaining data comprising both adult trees and regenerating cohorts. Henceforth, we therefore use forest regeneration as an umbrella term to collectively describe all the woody vegetation outcome responses. The papers reported on research conducted in 41 forest landscapes in 27 countries (Fig. 1). They consisted of ‘observed’ studies of real-world defaunation and ‘manipulated’ studies where faunal abundance was experimentally altered, generally through the use of vertebrate exclosures. In total, the papers generated 107 pairwise comparisons between a low fauna (typically hunted and/or fragmented forests, or vertebrate exclosures) and a high fauna treatment (such as contiguous and/or protected forests, or open controls in exclosure experiments). To quantify the impact of defaunation on forest regeneration we calculated woody vegetation outcome response effect sizes between the high and low fauna treatments. We carried out four analyses, grouping the data according to the (i) category of defaunated taxonomic group, (ii) type of woody vegetation-animal interaction disrupted by defaunation, (iii) geographic region and (iv) seed dispersal syndrome of regenerating trees.

We find that vertebrate defaunation has a significant negative impact on forest regeneration in observed studies of real-world defaunation, but a positive one when defaunation is experimentally manipulated. When only observed studies are considered, defaunation of primates and birds is deleterious to forest regeneration, as is the loss of seed dispersal services. Therefore, the conservation of intact faunal communities is imperative to ensure the composition and functioning of forests into the future.

## Results

### Defaunation impacts on regeneration

Vertebrate defaunation is associated with significant effects on forest regeneration, although the direction and magnitude of the impact depends on the methodological approach adopted in the study, category of taxonomic group, type of woody vegetation-animal interaction disrupted, geographic region, and seed dispersal syndrome. The overall effect of defaunation is negative and significantly different from zero for observed studies (mean Hedges’ *g* [± 95% CI] = −0.68 [−1.07 to −0.29], *p* = 0.001), yet positive and significant across manipulated ones (mean Hedges’ *g* [± 95% CI] = 0.45 [0.11–0.79], *p* = 0.01) (Supplementary Table 2). The mean effect sizes for observed and manipulated studies were significantly different from one another (Cochran’s *Q*_M_ = 15.21, *p* < 0.001). Thus the extirpation of vertebrates deleteriously influences forest regeneration in real-world contexts, although it has the opposite impact when induced experimentally.

**Fig. 2:**
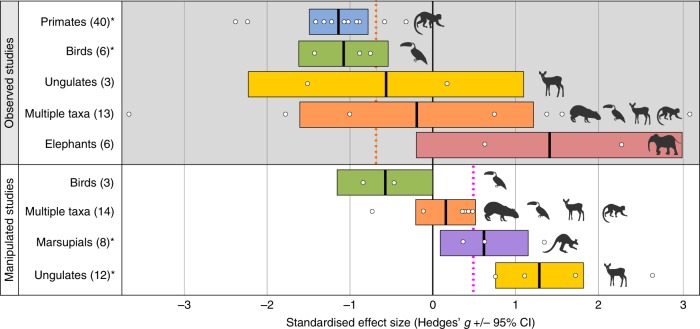
Effect sizes of defaunation on forest regeneration by taxonomic group category. Observed and manipulated studies are analysed separately. The number of pairwise comparisons between high fauna and low fauna treatments per category is reported in parentheses, mean standardised effect size (Hedges’ *g*) is indicated by the bold vertical line in the centre of the coloured box, and 95% confidence intervals are represented by the width of the coloured box. Asterisks (*) denote categories where the confidence intervals do not overlap zero, indicating a significant effect (Hedges’ *g*). Please note that the figure shows the impact related to the absence of each taxonomic group category; a negative effect size means that defaunation is having a detrimental impact on forest regeneration. Vertical dashed lines show the mean overall effect size for observed (−0.68) and manipulated (+0.45) studies, respectively

**Fig. 3:**
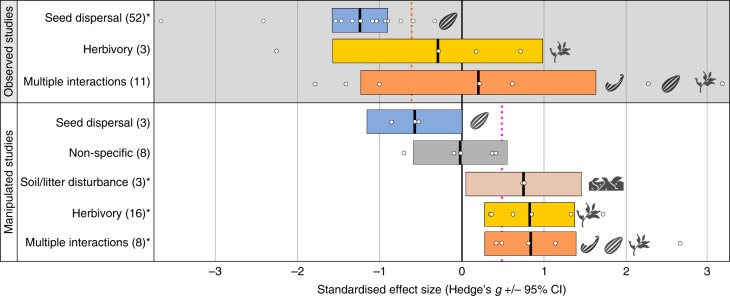
Effect sizes of defaunation on forest regeneration by woody vegetation-animal interaction type. Observed and manipulated studies are analysed separately. The number of pairwise comparisons between high fauna and low fauna treatments per category is reported in parentheses, mean standardised effect size (Hedges’ *g*) is indicated by the bold vertical line in the centre of the coloured box, and 95% confidence intervals are represented by the width of the coloured box. Asterisks (*) denote categories where the confidence intervals do not overlap zero, indicating a significant effect (Hedges’ *g*). Please note that the figure shows the impact related to the absence of each woody vegetation-animal interaction type; a negative effect size means that defaunation is having a detrimental impact on forest regeneration. Vertical dashed lines show the mean overall effect size for observed (−0.68) and manipulated (+0.45) studies, respectively

**Fig. 4:**
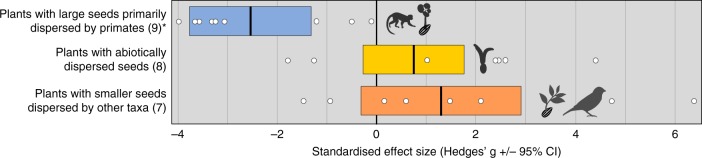
Effect sizes of defaunation on forest regeneration by seed dispersal syndrome. All studies were observed. The number of pairwise comparisons between high fauna and low fauna treatments per category is reported in parentheses, mean standardised effect size (Hedges’ *g*) is indicated by the bold vertical line in the centre of the coloured box, and 95% confidence intervals are represented by the width of the coloured box. Asterisks (*) denote categories where the confidence intervals do not overlap zero, indicating a significant effect (Hedges’ *g*). Please note that the figure shows the impact of defaunation on the regeneration of woody plants with different dispersal syndromes; a negative effect size means that defaunation is having a detrimental impact on regeneration of defined groups

The impacts of defaunation vary according to the category of taxonomic group investigated (Fig. 2). Defaunation of primates and birds leads to adverse impacts on forest regeneration in observed studies, whereas marsupial and ungulate population declines have positive impacts on regeneration in manipulated studies. In addition, the disruption of specific woody vegetation-animal interactions influences forest regeneration in different ways. Decreased seed dispersal reduces forest regeneration in observed systems, while diminished soil/litter disturbance and herbivory has positive impacts on regeneration in manipulated studies (Fig. 3). When the data are explored by geographic region, defaunation in the Neotropics, Asia and Australasia & Oceania has a significant adverse effect on forest regeneration in observed studies, yet the reduction of vertebrate populations in manipulation studies has the opposite effect in Europe and Australasia & Oceania (Supplementary Table 2). Finally, woody plants with large seeds that are primarily dispersed by primates suffer negative effects as a consequence of defaunation within observed studies (Fig. 4).

## Discussion

Forests have long been the focus of biodiversity conservation policies, but have become prioritised increasingly within sustainable development and climate change multilateral agreements over the last decade^1,21^. Numerous authors have suggested that the continued provision of forest ecosystem services that underpin human well-being will depend on the maintenance of intact ecological communities^1,20,22–27^. Nonetheless, our meta-analysis is the first to bring together the weight of evidence from local-scale studies conducted around the world, and demonstrates that, across a range of forest ecosystems, defaunation appears to alter forest regeneration and woody vegetation community composition. Moreover, we provide quantitative insights into the mechanisms and taxonomic groups driving these changes.

**Fig. 5:**
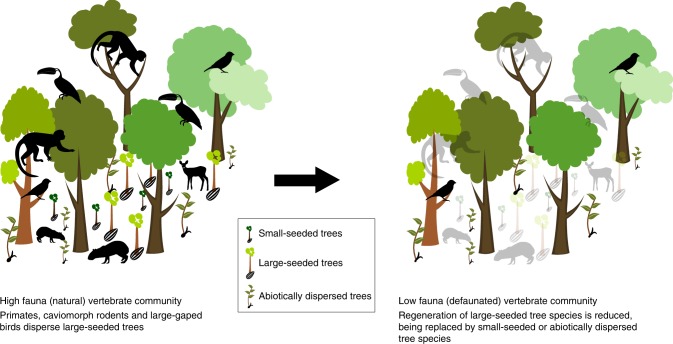
Schematic representation of forest regeneration in natural and defaunated communities

Defaunation-induced disruptions to forest regeneration are particularly concerning given that forests are among the largest terrestrial carbon stores on the planet^28,29^. Our meta-analysis shows that the extirpation of vertebrates causes changes to forest dynamics even in the absence of direct anthropogenic threats in the form of deforestation and structural degradation. While only one of the studies we examined measured the effect of defaunation on forest biomass or carbon storage directly^30^, the remaining papers reveal potential shifts in the functional trait composition of woody vegetation communities. Following the loss of vertebrates, there is a decline in regeneration of trees with large seeds that are primarily dispersed by primates, which appear to be replaced by tree species that are dispersed abiotically or by other smaller animals^19,31–36^ (Figs. 4 and 5). Given that small-seeded and abiotically dispersed trees are typically less carbon dense than large-seeded, animal-dispersed species^37–39^, the carbon store in the adult tree community is likely to be similarly reduced in the absence of primates and other large seed dispersing taxa. Some authors predict that the carbon storage potential of tropical forests will decrease by up to 38% if this happens^27^, although the impacts are expected to vary in different regions and forest types^20,26,40^. Our regional analysis substantiates this suggestion.

Deforestation and forest degradation are responsible for 7–17% of global carbon emissions^41,42^ and have contributed 26% of all emissions associated with anthropogenic activities since 1870^43^. Climate change policies therefore focus on decreasing forest loss through mechanisms such as REDD + (Reduced Emissions from Deforestation and forest Degradation, and forest conservation, sustainable management and enhancement of forest carbon stocks in developing countries). However, our results strengthen the widely made assertion that the success of forest-based climate mitigation initiatives is also contingent on the protection of vertebrate populations inhabiting them^1,20,22–27^. Specifically, vertebrates such as primates, large-gaped birds (e.g. toucans, hornbills) and caviomorph rodents, which are capable of dispersing large seeds, should be at the centre of conservation efforts^33,44–54^. Consequently, the maintenance and enhancement of biodiversity should not simply be perceived as a secondary co-benefit of REDD +, but as co-dependent and central to the delivery and resilience of the carbon storage benefit itself. Indeed, forest and climate strategies need to be alert not only to deforestation fronts, but also defaunation fronts that risk a long-term decline in forest carbon stores even if the canopy may appear intact. Moreover, our findings also suggest that the maintenance or re-establishment of intact faunal communities, with a particular focus on dispersers of large seeds, will be critical to the long-term success of ecological restoration efforts such as reforestation^55^, and reinforces calls for trophic rewilding as a conservation strategy to maintain ecosystem function^56^.

Critically, for translating evidence into policy, the results presented here lend support to the suggestion that the methodological approach used to investigate the impacts of defaunation greatly influences the direction of the effect^25,57^. Studies based on observations of real-world defaunation, primarily caused by hunting and fragmentation, show that the extirpation of vertebrates has negative overall impacts on forest regeneration. However, when vertebrates are excluded from forests in manipulation experiments, this artificial defaunation points to the opposite effect. This is because hunting/fragmentation and exclosures influence vertebrate communities in different ways^25,57^. Exclosures effectively keep out ground-dwelling vertebrates, which tend to be herbivores (e.g. ungulates) or seed predators (e.g. rodents), but do not always exclude arboreal and volant species such as primates, birds and bats^58–60^. Since the latter are often seed dispersers, exclosures serve to reduce seed and seedling predation without diminishing dispersal, leading to rises in seedling density and richness. However, hunting and fragmentation tend to affect dispersers as well as plant antagonists^19,61–63^, so real-world defaunation leads to overall decreases in seedling density and richness. Therefore, while exclosure experiments provide a useful approach for elucidating the effects of removing particular components of the vertebrate fauna, they should not be viewed as analogous to real-world defaunation^64^.

In conclusion, our study suggests that vertebrates play a critical role in forest regeneration, and that defaunation will thus have negative impacts on future forest community composition and the provision of ecosystem services such as carbon storage. Future climate change policymaking, planning, management and monitoring, including REDD + programmes, should explicitly incorporate measures to maintain vertebrate populations, in addition to forest cover, if they are to achieve their goals long term. This also serves to highlight the value of integrated approaches to delivering on United Nations Framework Convention on Climate Change (UNFCCC) and Convention on Biological Diversity (CBD) objectives nationally and internationally.

## Methods

### Literature search

We used a rapid evidence assessment (REA) approach^65,66^ to search for papers measuring woody vegetation responses associated with defaunation in forests, based on inclusion and exclusion criteria defined a priori. We examined all English language studies, conducted in any type of forest (as described by the study authors), without restrictions on publication date. We searched for papers published online by 28th February 2019 using Web of Science, applying a search string optimised through a scoping trial (Supplementary Table 3). In addition, we searched the reference lists of all relevant papers, plus key reviews conducted on defaunation^12,64,67–74^, to locate further pertinent studies. We sequentially screened the title, abstract and full text of each paper for relevance, taking a conservative approach to inclusion. A subset of studies was evaluated independently by two of the authors at each screening stage. The level of agreement between decisions to accept or reject material was assessed using Cohen’s kappa statistic^75^ and a threshold of >0.7, ensuring the inclusion/exclusion criteria were correctly and consistently applied.

Studies were included if they (i) were based on empirical (as opposed to modelled) data, (ii) used an experimental or quasi-experimental design to compare between treatments that differed in the abundance, density or richness of vertebrate faunas (e.g. exclosure vs. control, defaunated vs. non-defaunated); (iii) reported woody vegetation response data for all treatments; and (iv) provided variance measures for woody vegetation responses (or if these could be calculated from the published data). We included research from fragmented systems only when differences in faunal abundance between treatments were explicitly stated, and from systems undergoing both defaunation and logging only when the direct effects of logging (i.e. differences in adult tree density between logged and unlogged treatments) were controlled for. We excluded studies focusing on non-native vertebrates or livestock, plantation forests, herbs and non-native plants, as well as those reporting the effects of cervid hyperabundance in temperate regions (because these ecosystems are not experiencing defaunation).

The REA revealed 184 relevant studies (Supplementary Fig. 1), which reported two broad types of response data: (i) outcome responses (i.e. how woody vegetation responded to defaunation) and (ii) process responses (i.e. processes that lead to woody vegetation outcomes influenced by defaunation) (Supplementary Table 4). Woody vegetation outcome responses are the most informative for forest regeneration because they directly quantify how woody vegetation communities respond to defaunation, rather than quantifying change in the processes that contribute to those outcomes. As such, only studies reporting woody vegetation outcome responses were included in the meta-analysis. The methodological approach of the studies fell into one of two categories. The first were those where the low fauna treatment was ‘manipulated’ via the use of vertebrate exclosures, or, in two cases, the addition of bird perches (16 studies with 38 pairwise comparisons). The second were cases where the low fauna treatment was ‘observed’, resulting from hunting, fragmentation or other causes (27 studies with 69 pairwise comparisons). Our final dataset consisted of data derived from 43 papers (Supplementary Fig. 1), which yielded 107 pairwise comparisons between low fauna (defaunated or exclosure) and high fauna (non-defaunated or control; although these treatments did not always represent intact faunal communities) treatments.

### Meta-analysis

For each pairwise comparison, we extracted woody vegetation outcome response data (mean and standard deviation) from the high and low fauna treatments. Data were obtained from the text, tables and figures of each study: if the data were presented graphically, we used WebPlotDigitizer (https://automeris.io/WebPlotDigitizer/) to extract them. Where multiple different woody vegetation outcome response metrics (e.g. different plant species or cohorts), or independent study sites were reported separately in a study, the relevant data for each were extracted (resulting in a total of 107 pairwise comparisons). Sample sizes were the number of independent sites, as reported by the study authors. For each pairwise comparison, we also documented the category of taxonomic group (birds, elephants, marsupials, primates, rodents, ungulates, or multi-taxa), woody vegetation-animal interaction type (herbivory, pollination, seed dispersal, seed predation, soil/litter disturbance, or multiple interactions), geographic region (Africa, Neotropics, Australasia & Oceania, Asia, or Europe), seed dispersal syndrome (woody species with large, primarily primate-dispersed seeds, smaller seeds dispersed by other taxa, or abiotically dispersed seeds), and the methodological approach (observed or manipulated).

To assess the impact of defaunation on forest regeneration we calculated the Hedges’ *g* effect size of the standardised mean difference in woody vegetation outcome response metrics between all high and low fauna treatments. We used a fully random-effects model because we did not expect there to be one true effect size, due to the diversity of metrics used across the studies (e.g. different studies investigated different plant species, woody vegetation outcome response metrics, faunal species defaunated, defaunation cause/intensity and so on). The model thus accounted for two levels of error, weighting each study by the inverse of its variance, as well as the between-study variance^76–79^. Moreover, as most studies provided multiple pairwise comparisons, we accounted for the potential non-independence of these by nesting them within each study, computing a mean for each study^76,80^.

We did not calculate an overall mean effect size for all studies combined because of the opposing effect size directions for manipulated and observed studies, and because the heterogeneity between studies was significant (Cochran’s *Q*_M_ = 439, *p* < 0.001, *I*^2^ = 90%, *τ*^2^ = 0.86). Therefore, our first analysis consisted of two overall effect sizes for observed and manipulated studies separately. We defined the effect direction as negative for comparisons of mutualistic interactions (e.g. seed dispersal, pollination), because in these cases the woody vegetation outcome responses decreased in low fauna treatments, while in studies of antagonistic interactions (e.g. seed predation, herbivory) the woody vegetation outcome responses increased and so the effect direction was defined as positive. Therefore, a negative effect size indicates that defaunation is associated with reduced woody vegetation outcome response values, while the opposite is true for positive effect sizes. Effect sizes were considered significant if the confidence interval did not overlap zero^78,81^. After calculating the effect size for observed and manipulated studies, we conducted the meta-analysis across all levels of the moderator variables (category of taxonomic group, woody vegetation-animal interaction type, geographic region and seed dispersal syndrome).

To test our dataset for publication bias, we followed Nakagawa et al.^82^. We plotted two types of funnel plot and calculated the associated Kendall’s tau (Supplementary Fig. 2). As the plots were largely symmetrical, and the test was non-significant, we concluded that no such bias existed in our dataset. In addition, we calculated the Classic (Rosenthal’s) Fail-safe *N* which was 189, meaning that we would need to locate and include 189 studies with an effect size of zero in order to overturn the result^76,78^. Likewise, no evidence of temporal bias was apparent, on examination of a meta-regression of publication year against effect size, or a cumulative meta-analysis ordered by year (Supplementary Fig. 3). All analyses were conducted using the Comprehensive Meta-analysis software^83^.

### Reporting summary

Further information on research design is available in the Nature Research Reporting Summary linked to this article.

## Supplementary information

Supplementary Information

Reporting Summary

## Data Availability

The datasets generated and analysed during this study are open access and available in the University of Kent Academic Repository at http://data.kent.ac.uk/35/.
