# Supplementary material for: Quantifying the impacts of defaunation on natural forest regeneration in a global meta-analysis
Source: Nat Commun. 2019 Oct 14;10:4590. doi: 10.1038/s41467-019-12539-1 (PMC6791894; doi:10.1038/s41467-019-12539-1)
Supplement: Supplementary file 1 — Supplementary Information [file 41467_2019_12539_MOESM1_ESM.pdf]

Quantifying the impacts of defaunation on natural forest regeneration in a global meta-analysis

Gardner et al.

Supplementary Information

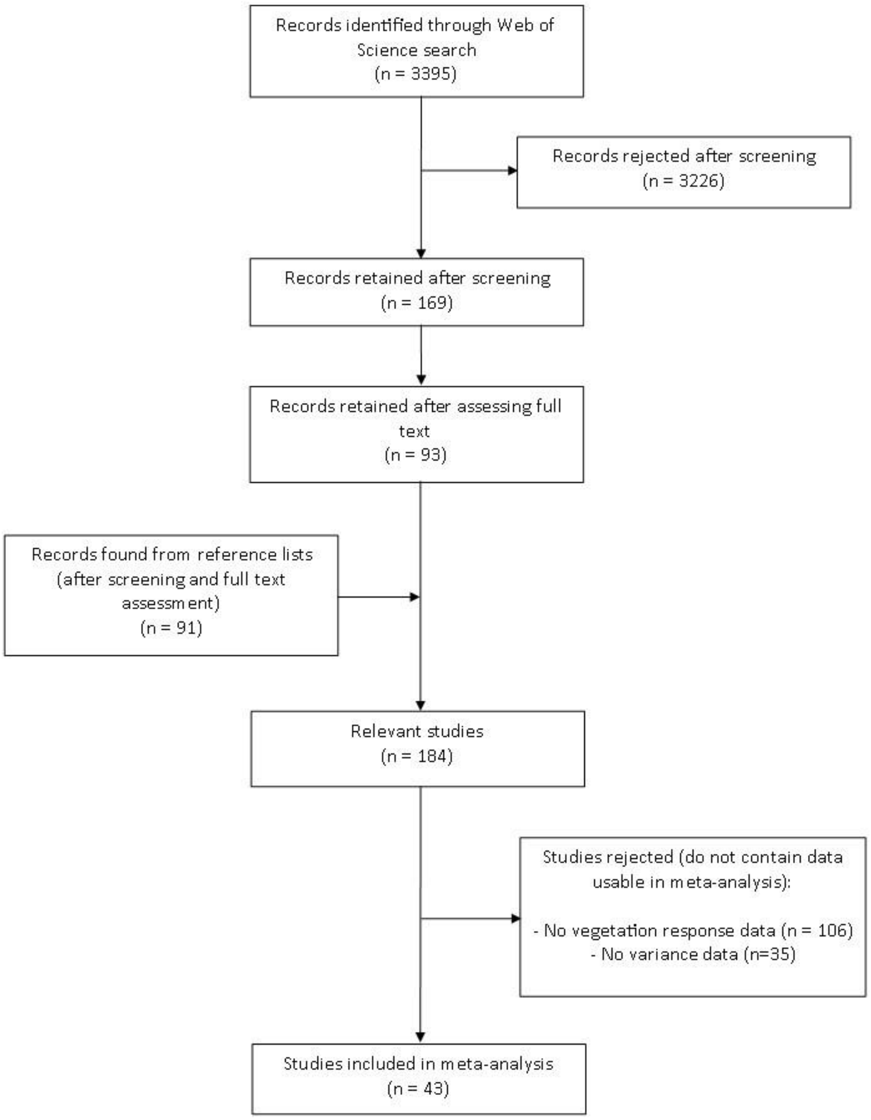

Supplementary Figure 1. The exclusion of studies at each filtering stage of the rapid evidence assessment and meta-analysis.

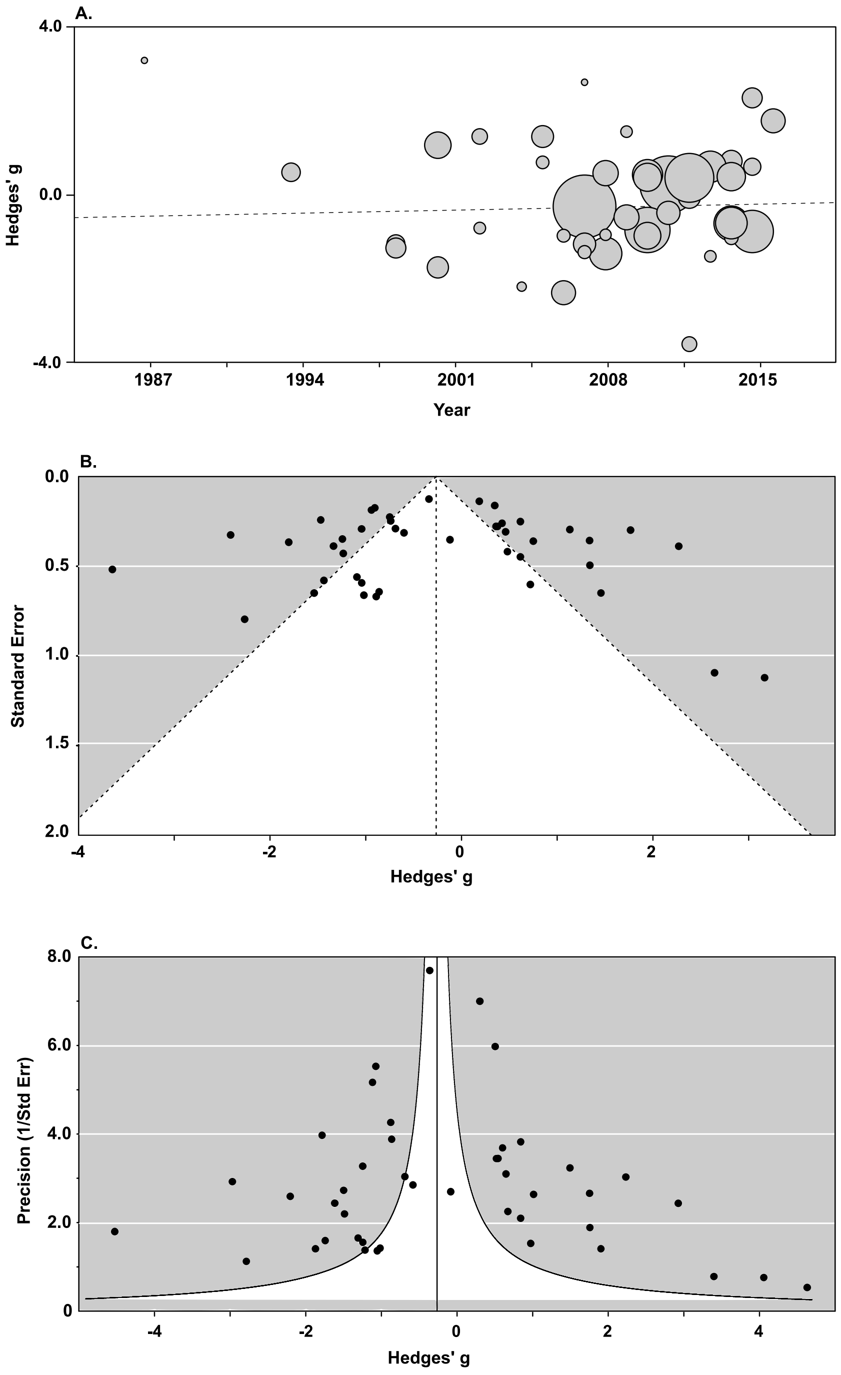

Supplementary Figure 2. Assessments of publication bias in the meta-analysis. A. Meta-regression of the mean effect size of each study against publication year (Q <0.001, p = 0.96). Circle size indicates the study precision, with more precise studies indicated by larger circles. B. Funnel plot of the relationship between the mean effect size and standard error for each study. C. Funnel plot of the relationship between the mean effect size and the precision of each study. Kendall's tau = 0.01, p = 0.92.

A.

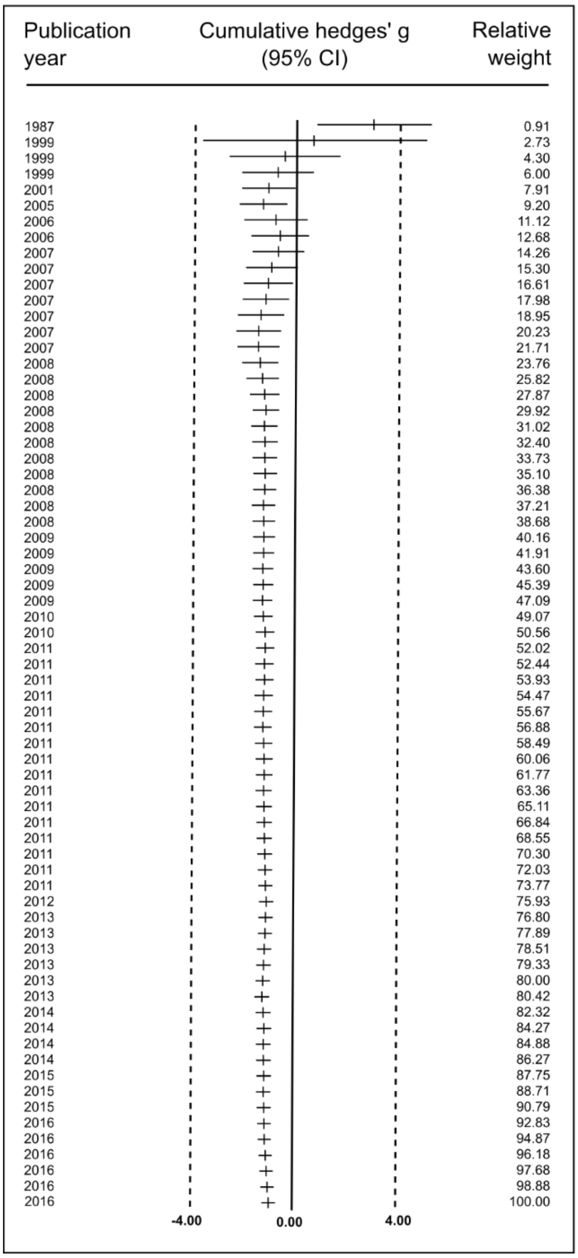

B.

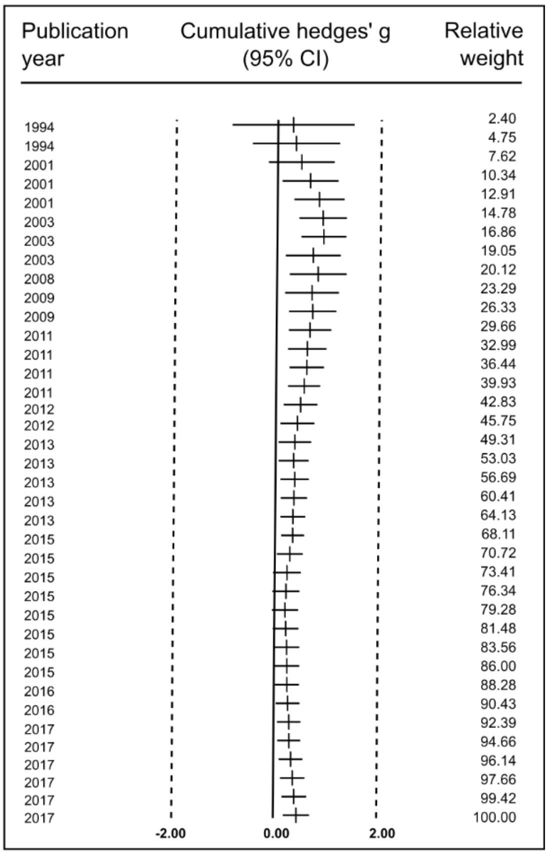

Supplementary Figure 3. Cumulative meta-analysis over time (ordered by publication year). A. Pairwise comparisons from observed defaunation studies B. Pairwise comparisons from manipulated defaunation studies.

Supplementary Table 1. The 43 papers examining woody vegetation outcome responses that were included in the meta-analysis of defaunation impacts on natural forest regeneration. The full dataset is available in the University of Kent Academic Repository at http://data.kent.ac.uk/35/.

Author and date	Title	Country	Study design	Defaunation cause	Fauna investigated	Interactions investigated
Anderson et al 2011[1]	Cascading effects of bird functional extinction reduce pollination and plant density	New Zealand	Observed	Mainland extinctions	Birds	Pollination
Anzures-Dadda et al 2011[2]	Absence of howlers (*Alouatta palliata*) influences tree seedling densities in tropical rain forest fragments in southern Mexico	Mexico	Observed	Not specified	Primates	Seed dispersal
Asquith et al 1999[3]	The fruits the agouti ate: *Hymenaea courbaril* seed fate when its disperser is absent	Venezuela	Observed	Fragmentation	Rodents	Seed removal, seed dispersal
Bongi et al 2017[4]	Wild boar impact on forest regeneration in the northern Apennines (Italy)	Italy	Manipulated		Ungulates	Herbivory
Brocardo et al 2013[5]	No changes in seedling recruitment when terrestrial mammals are excluded in a partially defaunated Atlantic rainforest	Brazil	Manipulated		Marsupials, rodents, armadillos, primates, carnivores, ungulates	Non-specific
Calle-Rendon et al 2016[6]	Comparison of forest regeneration in two sites with different primate abundances in Northwestern Ecuador	Ecuador	Observed	Hunting	Primates	Seed dispersal
Camargo-Sanabria et al 2015[7]	Experimental defaunation of terrestrial mammalian herbivores alters tropical rainforest understorey diversity	Mexico	Manipulated		Marsupials, rodents, armadillos, carnivores, ungulates	Non-specific
Chaves et al 2015[8]	Primate extirpation from rainforest fragments does not appear to influence seedling recruitment	Mexico	Observed	Fragmentation	Primates	Seed dispersal
Cramer et al 2007[9]	Forest fragmentation reduces seed dispersal of *Duckeodendron cestroides*, a Central Amazon endemic	Brazil	Observed	Fragmentation	Primates, rodents, ungulates	Seed dispersal

Dexter et al 2013[10]	Unintended consequences of invasive predator control in an Australian forest: overabundant wallabies and vegetation change	Australia	Manipulated		Marsupials	Herbivory
Effiom et al 2013[11]	Bushmeat hunting changes regeneration of African rainforests	Nigeria	Observed	Hunting	Primates, rodents, ungulates	Seed dispersal
Feeley & Terborgh 2005[12]	The effects of herbivore density on soil nutrients and tree growth in tropical forest fragments	Venezuela	Observed	Fragmentation	Primates	Herbivory
Foster et al 2016[13]	Herbivory and fire interact to affect forest understory habitat, but not its use by small vertebrates	Australia	Manipulated		Marsupials	Herbivory
Ganzhorn et al 1999[14]	Lemurs and the regeneration of dry deciduous forest in Madagascar	Madagascar	Observed	Fragmentation	Primates	Seed dispersal
Gomez & Holdar 2008[15]	Wild boars (*Sus scrofa*) affect the recruitment rate and spatial distribution of holm oak (*Quercus ilex*)	Spain	Manipulated		Ungulates	Herbivory, physical damage
Graham & Page 2012[16]	Artificial bird perches for the regeneration of degraded tropical peat swamp forest: a restoration tool with limited potential	Indonesia	Manipulated		Birds	Seed dispersal
Gutierrez-Granados & Dirzo 2010[17]	Indirect effects of timber extraction on plant recruitment and diversity via reductions in abundance of frugivorous spider monkeys	Mexico	Observed	Logging	Primates	Seed dispersal
Hanson et al 2006[18]	Variation in seedling density and seed predation indicators for the emergent tree *Dipteryx panamensis* in continuous and fragmented rainforest	Costa Rica	Observed	Hunting, fragmentation	Ungulates, rodents, birds	Seed predation
Ickes et al 2001[19]	Effects of native pigs (*Sus scrofa*) on woody understorey vegetation in a Malaysian lowland rain forest	Malaysia	Manipulated		Ungulates	Seed predation, soil/litter disturbance
Keuroghlian & Eaton 2009[20]	Removal of palm fruits and ecosystem engineering in palm stands by white-lipped peccaries (*Tayassu pecari*) and other frugivores in an isolated forest fragment	Brazil	Manipulated		Ungulates, rodents	Seed predation, herbivory

Kurten & Carson 2015[21]	Do ground-dwelling vertebrates promote diversity in a Neotropical forest? Results from a long-term exclosure experiment	Panama	Manipulated		Rodents, ungulates, birds	Non-specific
Lagendijk et al 2011[22]	The effects of herbivory by a mega- and mesoherbivore on tree recruitment in Sand forest, South Africa	South Africa	Manipulated		Elephants, ungulates	Herbivory
Lermyte & Forget 2009[23]	Rapid assessment of dispersal failure and seedling recruitment of large-seeded non-timber forest products trees in a tropical rainforest	French Guyana	Observed	Hunting, forest exploitation	Ungulates, primates, rodents	Seed removal, seed dispersal
Meers & Adams 2003[24]	The impact of grazing by eastern grey kangaroos (*Macropus giganteus*) on vegetation recovery after fire at Reef Hills Regional Park, Victoria	Australia	Manipulated		Marsupials	Herbivory
Michel et al 2015[25]	Do collared peccaries negatively impact understory insectivorous rain forest birds indirectly via lianas and vines?	Panama/ Costa Rica	Observed	Not specified	Ungulates	Soil/litter disturbance
Nuñez-Iturri & Howe 2007[26]	Bushmeat and the fate of trees with seeds dispersed by large primates in a lowland rain forest in Western Amazonia	Peru	Observed	Hunting	Primates	Seed dispersal
Nuñez-Iturri et al 2008[27]	Hunting reduces recruitment of primate-dispersed trees in Amazonian Peru	Peru	Observed	Hunting	Primates	Seed dispersal
O'Kane et al 2012[28]	Heavy impact on seedlings by the impala suggests a central role in woodland dynamics	South Africa	Observed	Natural variation	Ungulates	Herbivory
Pejchar 2015[29]	Introduced birds incompletely replace seed dispersal by a native frugivore	USA (Hawaii)	Observed	Local extinction	Birds	Seed dispersal
Roldan & Simonetti 2001[30]	Plant-mammal interactions in tropical Bolivian forests with different hunting pressures	Bolivia	Observed	Hunting	Ungulates, carnivores, primates	Seed removal, herbivory
Rutina & Moe 2014[31]	Elephant (*Loxodonta africana*) disturbance to riparian woodland: effects on tree species richness, diversity and functional redundancy	Botswana	Observed	Natural variation	Elephant	Herbivory, physical damage

Sethi & Howe 2009[32]	Recruitment of hornbill-dispersed trees in hunted and logged forests of the Indian Eastern Himalaya	India	Observed	Hunting	Birds	Seed dispersal
Shiels & Walker 2003[33]	Bird perches increase forest seeds on Puerto Rican landslides	Puerto Rico	Manipulated		Birds	Seed dispersal
Sica et al 2014[34]	Spatial pattern of pindo palm (*Syagrus romanzoffiana*) recruitment in Argentinian Atlantic Forest: the importance of tapir and effects of defaunation	Argentina	Observed	Fragmentation	Ungulates	Seed dispersal
Sork 1987[35]	Effects of predation and light on seedling establishment in *Gustavia superba*	Panama	Observed	Fragmentation	Ungulates, rodents	Seed predation, herbivory
Stevenson & Aldana 2008[36]	Potential effects of ateline extinction and forest fragmentation on plant diversity and composition in the western Orinoco Basin, Colombia	Colombia	Observed	Fragmentation	Primates	Seed dispersal
Stevenson 2011[37]	The abundance of large ateline monkeys is positively associated with the diversity of plants regenerating in Neotropical forests	Colombia/ Peru/ Ecuador	Observed	Hunting	Primates	Seed dispersal
Terborgh & Wright 1994[38]	Effects of mammalian herbivores on plant recruitment in two Neotropical forests	Panama/ Peru	Manipulated	Fragmentation	Rodents, ungulates, carnivores	Seed removal, herbivory
Terborgh et al 2006[39]	Vegetation dynamics of predator-free land-bridge islands	Venezuela	Observed	Fragmentation	Birds, rodents, primates, reptiles	Herbivory
Terborgh et al 2016[40]	Megafaunal influences on tree recruitment in African equatorial forests	Gabon	Observed	Hunting	Elephant	Herbivory, physical damage
Theimer et al 2011[41]	Terrestrial vertebrates alter seedling composition and richness but not diversity in an Australian tropical rain forest	Australia	Manipulated		Marsupials, birds	Non-specific
Vanthomme et al 2010[42]	Bushmeat hunting alters recruitment of large-seeded plant species in central Africa	Central African Republic	Observed	Hunting	Ungulates, primates, rodents, birds	Non-specific
Zambrano et al 2008[43]	Diversity of regenerating plants and seed dispersal in two canopy trees from Colombian Amazon forests with different hunting pressure	Colombia	Observed	Hunting	Primates	Seed removal, seed dispersal

Supplementary Table 2. Summary statistics for all effect sizes (where data were available) calculated for all moderators within observed and manipulated studies. Significant effect sizes are shown in bold. Effect sizes based on just a single pairwise comparison are not a true meta-analysis and the results should be interpreted accordingly.

Analysis	Mean Hedges' g	Lower 95% CI	Upper 95% CI	p (effect size)	Q_M, p
**Methodological approach**					**Q_M = 15.21, p <0.001**
**Observed studies**	**-0.68**	**-1.07**	**-0.29**	**<0.001**	
**Manipulated studies**	**0.45**	**0.11**	**0.79**	**<0.01**	
**Taxonomic group (Fig. 2)**					**Q_M = 46.09, p <0.001**
Observed studies					
Birds	**-1.07**	**-1.62**	**-0.51**	**<0.001**	
Elephants	1.42	-0.20	3.04	0.09	
Multiple taxa	-0.18	-1.60	1.24	0.80	
Primates	**-1.12**	**-1.50**	**-0.75**	**<0.001**	
Rodents (single study)	**-1.23**	**-2.08**	**-0.38**	**<0.01**	
Ungulates	-0.55	-2.23	1.12	0.52	
Manipulated studies					
Birds	-0.57	-1.15	0.02	0.06	
Marsupials	**0.63**	**0.09**	**1.17**	**<0.05**	
Multiple taxa	0.17	-0.21	0.54	0.39	
Ungulates	**1.30**	**0.76**	**1.84**	**<0.001**	
**Interaction type (Fig. 3)**					**Q_M = 49.72, p <0.001**
Observed studies					
Herbivory	-0.28	-1.57	1.00	0.67	
Multiple interactions	0.21	-1.23	1.65	0.78	
Non-specific (single study)	**1.46**	**0.18**	**2.74**	**<0.05**	
Pollination (single study)	-0.88	-2.20	0.43	0.19	
Seed dispersal	**-1.23**	**-1.58**	**-0.88**	**<0.001**	
Seed predation (single study)	**1.34**	**0.64**	**2.05**	**<0.001**	
Manipulated studies					
Herbivory	**0.83**	**0.27**	**1.40**	**<0.01**	
Multiple interactions	**0.84**	**0.27**	**1.41**	**<0.01**	
Non-specific	-0.01	-0.59	0.57	0.97	
Seed dispersal	-0.57	-1.15	0.02	0.06	
Soil/litter disturbance	**0.76**	**0.04**	**1.47**	**<0.05**	
**Geographic region**					**Q_M = 29.57, p <0.001**
Observed studies					

**Analysis**	**Mean Hedges' g**	**Lower 95% CI**	**Upper 95% CI**	**p (effect size)**	**Q_M, p**
Africa	-0.06	-1.22	1.11	0.92	
Asia	**-1.46**	**-1.94**	**-0.98**	**<0.001**	
Neotropics	**-0.85**	**-1.26**	**-0.45**	**<0.001**	
Australasia & Oceania	**-0.76**	**-1.18**	**-0.33**	**<0.001**	
Manipulated studies					
Africa	0.37	-0.18	0.92	0.19	
Asia	0.33	-1.26	1.93	0.68	
Europe	**1.80**	**1.18**	**2.42**	**<0.001**	
Neotropics	0.09	-0.38	0.57	0.70	
Australasia & Oceania	**0.50**	**0.19**	**0.81**	**<0.01**	
**Seed dispersal syndrome (Fig. 4)**					
Observed studies					
Plants with abiotically dispersed seeds	0.90	2.05	-0.25	0.12	
Plants with large seeds primarily dispersed by primates	**-2.38**	**-1.30**	**-3.46**	**<0.001**	
Plants with smaller seeds dispersed by other taxa	1.10	2.25	-0.22	0.11	

Supplementary Table 3. Search string used to find relevant literature on defaunation impacts on natural forest regeneration via Web of Science. The different components of the string were used in parentheses and joined using the Boolean operator ‘AND’. An asterisk (*) denotes a wildcard.

Component 1	Component 2	Component 3	Component 4	Component 5
forest	*carbon* OR biomass OR vegetation OR tree	mammal OR primate OR monkey OR ape OR rodent OR bat OR carnivor* OR ungulate OR marsupial OR bird OR parrot OR reptile OR tortoise OR hunt* OR defaunat*	dispers* OR frugivor* OR pollinat* OR herbivor* OR graz* OR disturb* OR regenerat* OR recruit* OR seed*	exclu* OR experiment* OR plot OR simulat* OR model* OR investigat*

Supplementary Table 4. Response variables reported by relevant studies in the rapid evidence assessment that empirically investigated defaunation impacts on forests. Woody vegetation outcome responses measure change in woody vegetation variables following defaunation. Process responses merely quantify change in the processes that contribute to woody vegetation outcomes and, as such, only the woody vegetation outcome responses were included in the meta-analysis.

**Woody vegetation outcome responses**	
**Variable**	**Description**
Community density	Density of regenerating community of seedlings/saplings
Community richness	Species richness of regenerating community of seedlings/saplings
Species density	Density of regenerating seedlings/saplings of individual tree species
Species dispersion	Dispersion away from adult conspecifics of regenerating seedlings/saplings of individual tree species
Vegetation cover	Area coverage of forest or understory vegetation
Biomass	Above- or below-ground biomass of forest vegetation
Dispersal syndrome	Composition of the regenerating community of seedlings/saplings by dispersal syndrome (i.e. trees with large seeds primarily dispersed by primates, trees with smaller seeds dispersed by other taxa, abiotically-dispersed trees)
**Process responses**	
**Variable**	**Description**
Dispersal rate	Percentage of fruit/seeds successfully dispersed (i.e. removed but not predated) when seed fate is known
Dispersal distance	Mean dispersal distance of dispersed fruit/seeds
Seed rain	Species richness or density of aerially dispersed seeds caught in seed traps
Seed movement	Mean distance moved by seeds in non-trophic interactions (soil/litter disturbance)
Seed removal	Percentage of fruit/seeds removed when seed fate is unknown
Seed predation	Percentage of fruit/seeds predated
Germination rate	Germination rate from experimental seed placement
Seedling survival	Percentage of seedlings surviving
Seedling growth	Mean growth rate of seedlings
Leaf damage	Percentage of plants or leaves damaged by vertebrate herbivory
Arthropod herbivory rate	Percentage damage frequency or area of leaf damage caused by arthropod herbivory

Supplementary references

1. Anderson, S. H., Kelly, D., Ladley, J. J., Molloy, S. & Terry, J. Cascading effects of bird functional extinction reduce pollination and plant density. *Science* **331**, 1068–1071 (2011).
2. Anzures-Dadda, A., Andresen, E., Martinez, M. L. & Manson, R. H. Absence of howlers (*Alouatta palliata*) influences tree seedling densities in tropical rain forest fragments in southern Mexico. *Int. J. Primatol.* **32**, 634–651 (2011).
3. Asquith, N. M., Terborgh, J., Arnold, A. E., Riveros, N. & Carolina, N. The fruits the agouti ate: *Hymenaea courbaril* seed fate when its disperser is absent. *J. Trop. Ecol.* **15**, 229–235 (1999).
4. Bongi, P., Tomaselli, M., Petraglia, A., Tintori, D. & Carbognanai, M. Wild boar impact on forest regeneration in the northern Apennines (Italy). *Forest Ecol. Manag.* **391**, 230–238 (2017).
5. Brocardo, C. R., Zipparro, V. B., de Lima, R. A. F., Guevara, R. & Galetti, M. No changes in seedling recruitment when terrestrial mammals are excluded in a partially defaunated Atlantic rainforest. *Biol. Conserv.* **163**, 107–114 (2013).
6. Calle-Rendon, B. R., Peck, M., Bennett, S. E., Morelos-Juarez, C. & Alfonso, F. Comparison of forest regeneration in two sites with different primate abundances in Northwestern Ecuador. *Rev. Biol. Trop.* **64**, 493–506 (2016).
7. Camargo-Sanabria, A. A. et al. Experimental defaunation of terrestrial mammalian herbivores alters tropical rainforest understorey diversity. *Proc. Royal Soc. B* **282**, 20142580 (2015).
8. Chaves, O. M., Arroyo-Rodriguez, V., Martinez-Ramos, M. & Stoner, K. E. Primate extirpation from rainforest fragments does not appear to influence seedling recruitment. *Am. J. Primatol.* **77**, 468–478 (2015).
9. Cramer, J. M., Mesquita, R. C. G., Bentos, T. V., Moser, B. & Williamson, G. B. Forest fragmentation reduces seed dispersal of *Duckeodendron cestroides*, a Central Amazon endemic. *Biotropica* **39**, 709–718 (2007).
10. Dexter, N., Hudson, M., James, S., MacGregor, C. & Lindenmayer, D. B. Unintended consequences of invasive predator control in an Australian forest: overabundant wallabies and vegetation change. *PLoS ONE* **8**, e69087 (2013).
11. Effiom, E. O., Nuñez-Iturri, G., Smith, H. G., Ottosson, U. & Olsson, O. Bushmeat hunting changes regeneration of African rainforests. *Proc. Royal Soc. B* **280**, 20130246 (2013).
12. Feeley, K. J. & Terborgh, J. W. The effects of herbivore density on soil nutrients and tree growth in tropical forest fragments. *Ecology* **86**, 116–124 (2005).
13. Foster, C. N. et al. Herbivory and fire interact to affect forest understory habitat, but not its use by small vertebrates. *Anim. Conserv.* **19**, 15–25 (2016).
14. Ganzhorn, J. U., Fietz, J., Rakotovao, E., Schwab, D. & Zinner, D. Lemurs and the regeneration of dry deciduous forest in Madagascar. *Conserv. Biol.* **13**, 794–804 (1999).
15. Gomez, J. M. & Hodar, J. A. Wild boars (*Sus scrofa*) affect the recruitment rate and spatial distribution of holm oak (*Quercus ilex*). *Forest Ecol. Manag.* **256**, 1384–1389 (2008).

16. Graham, L. L. B. & Page, S. E. Artificial bird perches for the regeneration of degraded tropical peat swamp forest: a restoration tool with limited potential. *Restor. Ecol.* **20**, 631–637 (2012).
17. Gutierrez-Granados, G. & Dirzo, R. Indirect effects of timber extraction on plant recruitment and diversity via reductions in abundance of frugivorous spider monkeys. *J. Trop. Ecol.* **26**, 45–52 (2010).
18. Hanson, T., Brunsfeld, S. & Finegan, B. Variation in seedling density and seed predation indicators for the emergent tree *Dipteryx panamensis* in continuous and fragmented rainforest. *Biotropica* **38**, 770–774 (2006).
19. Ickes, K., Dewalt, S. J. & Appanah, S. Effects of native pigs (*Sus scrofa*) on woody understorey vegetation in a Malaysian lowland rain forest. *J. Trop. Ecol.* **17**, 191–206 (2001).
20. Keuroghlian, A. & Eaton, D. P. Removal of palm fruits and ecosystem engineering in palm stands by white-lipped peccaries (*Tayassu pecari*) and other frugivores in an isolated forest fragment. *Biodivers. Conserv.* **18**, 1733–1750 (2009).
21. Kurten, E. L. & Carson, W. P. Do ground-dwelling vertebrates promote diversity in a Neotropical forest? Results from a long-term exclosure experiment. *Bioscience* **65**, 862–870 (2015).
22. Lagendijk, D. D. G., Mackey, R. L., Page, B. R. & Slotow, R. The effects of herbivory by a mega- and mesoherbivore on tree recruitment in sand forest, South Africa. *PLoS ONE* **6**, e17983 (2011).
23. Lermyte, C. & Forget, P. -M. Rapid assessment of dispersal failure and seedling recruitment of large-seeded non-timber forest products trees in a tropical rainforest. *Trop. Conserv. Sci.* **2**, 404–424 (2009).
24. Meers, T. & Adams, R. The impact of grazing by eastern grey kangaroos (*Macropus giganteus*) on vegetation recovery after fire at Reef Hills Regional Park, Victoria. *Ecol. Manag. Restor.* **4**, 126–132 (2003).
25. Michel, N. L., Sherry, T. W. & Carson, W. P. The omnivorous collared peccary negates an insectivore-generated trophic cascade in Costa Rican wet tropical forest understorey. *J. Trop. Ecol.* **30**, 1–11 (2014).
26. Nuñez-Iturri, G. & Howe, H. F. Bushmeat and the fate of trees with seeds dispersed by large primates in a lowland rain forest in Western Amazonia. *Biotropica* **39**, 348–354 (2007).
27. Nuñez-Iturri, G., Olsson, O. & Howe, H. F. Hunting reduces recruitment of primate-dispersed trees in Amazonian Peru. *Biol. Conserv.* **141**, 1536–1546 (2008).
28. O'Kane, C. A. J., Duffy, K. J., Page, B. R. & Macdonald, D. W. Heavy impact on seedlings by the impala suggests a central role in woodland dynamics. *J. Trop. Ecol.* **28**, 291–297 (2012).
29. Pejchar, L. Introduced birds incompletely replace seed dispersal by a native frugivore. *AOB Plants* **7**, plv072 (2015).
30. Roldan, A. I. & Simonetti, J. A. Plant-mammal interactions in tropical Bolivian forests with different hunting pressures. *Conserv. Biol.* **15**, 617–623 (2001).
31. Rutina, L. P. & Moe, S. R. Elephant (*Loxodonta africana*) disturbance to riparian woodland: effects on tree species richness, diversity and functional redundancy. *Ecosystems* **17**, 1384–1396 (2014).

32. Sethi, P. & Howe, H. F. Recruitment of hornbill-dispersed trees in hunted and logged forests of the Indian Eastern Himalaya. *Conserv. Biol.* **23**, 710–718 (2009).
33. Shiels, A. B. & Walker, L. R. Bird perches increase forest seeds on Puerto Rican landslides. *Restor. Ecol.* **11**, 457–465 (2003).
34. Sica, Y. V., Bravo, S. P. & Giombini, M. I. Spatial pattern of pindo palm (*Syagrus romanzoffiana*) recruitment in Argentinian Atlantic Forest: the importance of tapir and effects of defaunation. *Biotropica* **46**, 696–703 (2014).
35. Sork, V. L. Effects of predation and light on seedling establishment in *Gustavia superba*. *Ecology* **68**, 1341–1350 (1987).
36. Stevenson, P. R. & Aldana, A. M. Potential effects of ateline extinction and forest fragmentation on plant diversity and composition in the western Orinoco Basin, Colombia. *Int. J. Primatol.* **29**, 365–377 (2008).
37. Stevenson, P. R. The abundance of large ateline monkeys is positively associated with the diversity of plants regenerating in Neotropical forests. *Biotropica* **43**, 512–519 (2011).
38. Terborgh, J. & Wright, S. J. Effects of mammalian herbivores on plant recruitment in two Neotropical forests. *Ecology* **75**, 1829–1833 (1994).
39. Terborgh, J., Feeley, K., Silman, M., Nuñez, P. & Balukjian, B. Vegetation dynamics of predator-free land-bridge islands. *J. Ecol.* **94**, 253–263 (2006).
40. Terborgh, J. et al. Megafaunal influences on tree recruitment in African equatorial forests. *Ecography* **39**, 180–186 (2016).
41. Theimer, T. C., Gehring, C. A., Green, P. T. & Connell, J. H. Terrestrial vertebrates alter seedling composition and richness but not diversity in an Australian tropical rain forest. *Ecology* **92**, 1637–1647 (2011).
42. Vanthomme, H., Belle, B. & Forget, P. -M. Bushmeat hunting alters recruitment of large-seeded plant species in central Africa. *Biotropica* **42**, 672–679 (2010).
43. Zambrano, V. A. B., Moncada, J. Z. & Stevenson, P. R. Diversity of regenerating plants and seed dispersal in two canopy trees from Colombian Amazon forests with different hunting pressure. *Rev. Biol. Trop.* **56**, 1531–1542 (2008).
